# Immunosuppressive Role of Integrin β8 in Recurrence After Bacillus Calmette–Guérin (BCG) Therapy for Non-Muscle Invasive Bladder Cancer

**DOI:** 10.3390/cancers17243964

**Published:** 2025-12-12

**Authors:** Seiji Hoshi, Satoru Meguro, Syunta Makabe, Akifumi Onagi, Emina Kayama, Kei Yaginuma, Hitomi Imai, Ryo Tanji, Ruriko Honda-Takinami, Kanako Matsuoka, Junya Hata, Yuichi Sato, Hidenori Akaihata, Soichiro Ogawa, Motohide Uemura, Yoshiyuki Kojima

**Affiliations:** Department of Urology, Fukushima Medical University School of Medicine, 1 Hikarigaoka, Fukushima 960-1295, Japan; s-meguro@fmu.ac.jp (S.M.);

**Keywords:** bladder cancer, immune response, immunosuppression, integrin β8 (ITGB8), TGF-β1, immune escape

## Abstract

**Background:** Although immunosuppressive factors contribute to the recurrence of non-muscle invasive bladder cancer (NMIBC) during Bacillus Calmette–Guérin (BCG) therapy, the mechanism remains unclear. **Methods:** In this study, key players in the immunosuppression associated with recurrence after BCG therapy were identified through comprehensive genetic and functional analyses of untreated NMIBC. **Results:** Microarray analysis of bladder cancer tissue from 13 eligible NMIBC patients revealed an association between high expression of *integrin β8* (*ITGB8*) and recurrence after BCG treatment (*p* < 0.001). In UMUC cells exhibiting high ITGB8 expression, *ITGB8* knockdown significantly decreased cell growth, invasion, adhesion, and secretion of the immunosuppressive cytokine transforming growth factor-β1 (TGF-β1, *p* < 0.01 for each). In addition, ITGB8 and TGF-β1 expression were correlated in immunohistochemistry of surgical specimens from 13 patients (*p* < 0.05). These results indicate that ITGB8 regulates TGF-β1 secretion. **Conclusions:** ITGB8 may contribute to post-BCG therapy recurrence of NMIBC by suppressing tumor immunity through the activation of TGF-β1. Evaluation of *ITGB8* expression in preoperative NMIBC could potentially predict recurrence following intravesical BCG therapy.

## Simple Summary

Patients with non-muscle invasive bladder cancer often receive Bacillus Calmette–Guérin (BCG) therapy to prevent cancer from returning. However, some patients still experience recurrence, and the reasons for this are not fully understood. This study investigated a molecule called integrin β8, which is found on the surface of cancer cells and may weaken the immune response activated by BCG treatment. We analyzed patient tumor samples and bladder cancer cells in the laboratory and found that tumors with high integrin β8 expression also showed increased levels of a substance that suppresses immunity. Patients with higher integrin β8 levels were more likely to have cancer return after BCG therapy. These findings suggest that integrin β8 may help cancer cells escape the immune system and could serve as a useful marker to identify patients at higher risk of recurrence before starting BCG treatment.

## 1. Introduction

Bladder cancer (BCa) is the ninth most common malignant disease worldwide, and 75% of BCa are non-muscle invasive bladder cancer (NMIBC), which does not invade the muscle layer [1]. In cases of high-risk NMIBC, administration of the Bacillus Calmette–Guérin (BCG) vaccine via intravesical infusion is effective for the prevention of intravesical recurrence and progression of muscle-invasive bladder cancer (MIBC) and remains the gold-standard treatment [2,3]. However, as high-risk patients have a 15-61% probability of intravesical recurrence [4] and 9.8% probability of progression to MIBC [5], BCG non-responders should be identified as early as possible. Furthermore, due to the greater likelihood of side effects compared with intravesical chemotherapy [5,6], some patients discontinue intravesical BCG therapy. This has resulted in increased demand for methods that can predict the effectiveness of BCG. However, the decision to administer BCG for NMIBC depends on clinical features, but it is difficult to predict the effectiveness of BCG before treatment. Thus, a noninvasive, simple, and low-cost method for identifying BCG non-responders is needed.

Over the short-term, BCG is thought to migrate into the bladder mucosa and subsequently exert anti-tumor effects either directly through cytotoxicity or indirectly as a result of invasion by immune cells in response to BCG-induced inflammation [7,8]. Over the mid- to long-term, by comparison, cells infected BCGs induce and sustain anti-tumor immunity in the bladder, thus contributing to the inhibition of BCa recurrence and progression [7,8]. These results indicate that immune induction is necessary for the anti-tumor effect of BCG in preventing recurrence and progression, with immune escape playing a role in the mid- to long-term resistance mechanism. Immune escape is comprehensively related to the induction of regulatory T cells expressing immunosuppressive molecules and suppressive cytokines [9,10,11,12,13]. However, the “trigger” molecules that initiate immune escape in NMIBC have not been identified.

Adhesion molecules have attracted considerable recent attention as regulators of the tumor microenvironment and immune response [14], and are located on the cell surface and make first contact at cell–cell and cell–extracellular matrix (ECM) junctions, thereby “triggering” a multitude of signaling pathways. We hypothesized that the therapeutic efficacy of BCG against NMIBC over the mid- to long term is associated with an immune escape mechanism induced by adhesion molecules in BCa cells.

In this study, we initially sought to identify the adhesion molecules associated with mid- to long-term intravesical recurrence using clinical specimens from high-risk NMIBC patients who have had BCG treatment. We then analyzed the function of the identified adhesion molecules and their relationship to immunosuppression in human bladder cell lines and clinical specimens. The overall purpose of this study was to discover novel target factors that could aid in the diagnosis of NMIBC and identify patients who are refractory to BCG treatment.

## 2. Materials and Methods

### 2.1. Patient and Surgical Specimens

**Figure 1:**
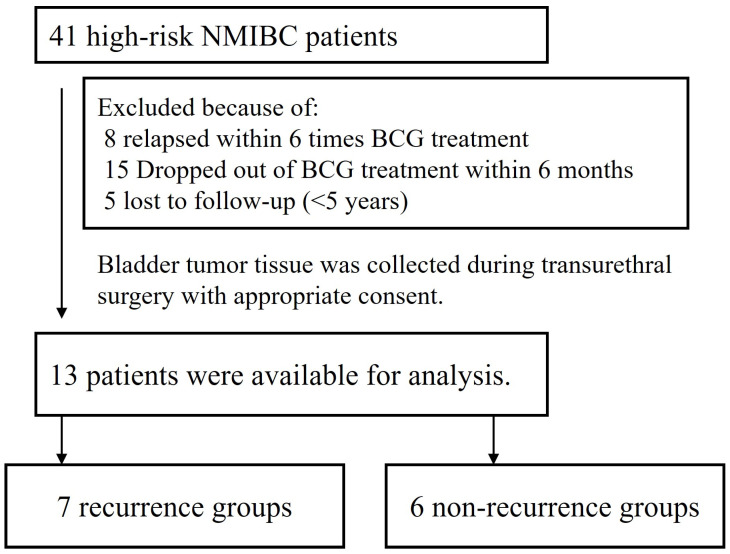
Selection and Exclusion of Patients in the Study. A total of 41 new-onset NMIBC patients were included in this study. Twenty-eight patients were excluded due to recurrence following induction of BCG therapy (8 patients), dropping out of BCG therapy (15 patients), and loss to follow-up (5 patients). The remaining 13 eligible patients were divided into two groups based on recurrence.

The study included 41 patients with high-risk NMIBC who were newly diagnosed and underwent transurethral resection of bladder tumor (TURBT) at our institution between 2014 and 2019. A total of 8 patients who experienced recurrence during BCG induction therapy (6 or fewer cycles) were excluded from the study in order to rule out major tumor residuals due to TURBT or multiple tumors. To avoid inclusion of patients who received inadequate BCG therapy, 15 patients who did not continue treatment according to the Southwest Oncology Group protocol [15] (10 patients discontinued due to side effects, and 5 patients discontinued due to refusal to continue treatment within 6 or fewer cycles) for at least 1 year and 5 patients who were untraceable were also excluded. These patients were excluded because inadequate BCG exposure and discontinuation due to excessive BCG-related adverse events could introduce immunological noise unrelated to tumor-derived biology. Eligible patients underwent routine cystoscopy and cytology and were confirmed to have had their tumors disappear at least once. Patients were observed for 5 years and divided into two groups based on recurrence or non-recurrence (Figure 1). Recurrence was defined as histologically confirmed urothelial carcinoma detected during follow-up cystoscopy and TURBT. Bladder tissue from 6 patients with suspected BCa who subsequently underwent mucosal biopsies that revealed no BCa was used as a control for the microarray analysis. Clinical information for the excluded 28 patients, including tumor characteristics is summarized in Supplementary Table S1.

Tumor tissue was collected with appropriate consent at the time of initial TURBT. Specimens used for microarray analysis were biopsied from the tumor surface before thermal denaturation and immediately frozen and stored until use. Specimens used for immunohistochemistry were fixed in formalin and then embedded in paraffin.

### 2.2. Microarray Analysis

Frozen specimens were used for total RNA extraction (Isogen, Nippon Gene, Tokyo, Japan) and poly(A)+ RNA purification (MicroPoly(A) Purist kit, Ambion, Austin, TX, USA). For DNA microarray analysis, 80-mer synthetic polynucleotides representing 14,400 human transcripts (MicroDiagnostic, Tokyo, Japan) were printed on glass slides. Labeled cDNA was synthesized from 5 μg total RNA using SuperScript II (Invitrogen, Carlsbad, CA, USA) with Cy5-dUTP (Perkin-Elmer, Waltham, MA, USA); reference cDNA was synthesized from Human Universal Reference RNA Type II (MicroDiagnostic) with Cy3-dUTP. Hybridization (MicroDiagnostic kit) was followed by scanning with a GenePix 4000B (Axon, San Francisco, CA, USA). Expression ratios (Cy5/Cy3) were normalized using GenePix Pro 3.0, converted to log2 values, and further processed with Microsoft Excel (Microsoft, WA, USA) and MDI software v3.0. (MicroDiagnostic). Expression levels were calculated relative to normal bladder tissue. Differential gene expression analysis was performed with *p*-values adjusted for multiplehypothesis testing using the Benjamini–Hochberg false discovery rate procedure. Genes with an adjusted *p*-value < 0.05 were considered statistically significant.

### 2.3. Reagents and Cell Lines

The human BCa cell lines RT4, RT24, RT112, and UMUC5 were utilized in this study. The cell lines were purchased from the American Type Culture Collection (ATCC, Altadena, CA, USA) and maintained in RPMI-1640 medium supplemented with 10% fetal bovine serum (cd-FBS) for experiments. Cells were grown at 37 °C in a humidified 5% CO_2_ environment. The medium was changed every 2 days, and cultures were split once each week.

### 2.4. cDNA Construction and Quantitative RT-PCR

cDNA was synthesized using a SuperPrep Cell Lysis and RT kit for qPCR (Toyobo, Tokyo, Japan) at 24 h after treatments. PCR reagents for ITGB8 (Hs00174456_m1) were purchased from Applied Biosystems (Waltham, MA, USA) following the manufacturer’s protocol. Quantitative RT-PCR assays were carried out using PowerTrack SYBR^®^ Green Master Mix (Invitrogen) on a StepOne real-time PCR system (Applied Biosystems). Gene expression data were standardized against β-actin gene expression using β-actin control reagent (Applied Biosystems).

### 2.5. Western Blotting Analysis

Protein extraction was carried out 24 or 72 h after drug exposure. Cells were lysed in RIPA buffer supplemented with protease inhibitors (Nacalai Tesque, Tokyo, Japan), and the resulting lysates were separated by SDS-PAGE. An anti-ITGB8 antibody (ab243023, Abcam, Waltham, MA, USA) was used as the primary antibody, and an anti β-actin antibody (Sigma-Aldrich Co., St. Louis, MO, USA) was used as an internal control. Protein bands were visualized using SuperSignal West Dura Extended Duration Substrate (Thermo Scientific, Waltham, MA, USA) and imaged using a ChemiDoc XRS plus system (Bio-Rad, Hercules, CA, USA). Individual bands were quantified using Image Lab 3.0 software (Bio-Rad) and normalized against the control value.

### 2.6. siRNA and Transfection

Silencer™ Select Pre-Designed siRNA was used for ITGB8 (s7599, Invitrogen) and the negative control (Silencer™ Select Negative Control No. 1 siRNA). For siRNA transfection, Lipofectamine RNAiMAX (Invitrogen) was applied as recommended by the manufacturer. One day before transfection, cells were seeded without antibiotics to reach 60–80% confluence. siRNA–Lipofectamine complexes were prepared in Opti-MEM medium (Thermo Scientific) by combining the siRNA oligomers with the reagent. The transfected cells were incubated for 24 h before subsequent treatments.

### 2.7. Adhesion Assay

RT4 and UMUC5 cells were knocked down before use in the experiment. A CytoSelect 48-well ECM Cell Adhesion Assay, ECM array kit (Cell Biolabs, San Diego, CA, USA) was used according to the manufacturer’s protocol. Cells were collected after knockdown and incubated for 1 h in serum-free medium to reach a density of 1 × 10^5^ cells/well. Cells adhering to the plate were lysed and stained, and the optical density at 560 nm was measured after extraction. A calibration curve was generated and used to determine the number of adherent cells.

### 2.8. ELISA

RT4 and UMUC5 cells were knocked down before use in the experiment. Cells were collected after knockdown and incubated for 1 h in serum-free medium to reach a density of 1 × 10^5^ cells/well. Knocked-down cells were cultured for 24 h in BioCoat^TM^ Fibronectin 48-well plates (Cosmo Bio Co., LTD. Tokyo, Japan) in serum-free medium, and the supernatant was collected for determination of the concentration of TGF-β1 using an ELISA kit (Cosmo Bio).

### 2.9. Immunohistochemistry

Paraffin-embedded sections of human BCa tissue were prepared and incubated with anti-ITGB8 (ab243023, Abcam), anti-TGF-β1 (ab92486, Abcam), and anti-TGF-β1 LAP-D (Cosmo Bio) primary antibodies. Subsequently, the sections were incubated with appropriate biotinylated secondary antibodies. Staining was detected using a streptavidin-biotin kit (Nichirei, Tokyo, Japan). We clarified that 3,3′ Diaminobenzidine (DAB) staining intensity was evaluated using a three-tier scale, which is widely accepted for cytokine-related markers; 0 = negative, 1+ weakly positive and 2+ positive. To improve objectivity and reproducibility, all immunohistochemistry slides were digitally analyzed using QuPath (version 0.6.0). All slides and QuPath outputs were independently reviewed by two urologists with extensive experience in pathological evaluation of urologic tumors.

Before automated quantification, the two evaluators confirmed and fine-tuned the DAB optical density thresholds within QuPath to ensure that the categorization into 0, 1+, and 2+ accurately reflected the biological staining differences. These thresholds were then uniformly applied to all samples in batch processing. The mean value of the five randomly selected fields was used as the final quantitative score and positive area for each marker. For quantitative area measurements, the final value was calculated as the average of the two observers’ measurements and for intensity scores (0/1+/2+) when the two observers assigned different scores, the final intensity score was determined through consensus discussion.

### 2.10. Statistical Analysis

Western blotting experiments, ELISAs, and analyses of cell proliferation, adhesion, invasion, and mRNA expression levels were repeated at least three times independently. Continuous variables are reported as the mean ± standard deviation (SD), and differences were evaluated using the unpaired Student’s *t*-test (two-tailed). Differences in categorical variables were analyzed using chi-square statistics. For nonparametric data, the Mann–Whitney U test was applied. Differences in recurrence-free survival was evaluated using Kaplan–Meier curves, and differences were assessed using the log–rank test. Differences were considered statistically significant at *p* < 0.05. All statistical analyses were performed using SPSS Statistics 21 software (IBM, Tokyo, Japan).

## 3. Results

### 3.1. Upregulation of the Adhesion Molecule Integrin β8 in BCG-Treated Patients with Recurrent NMIBC

**Figure 2:**
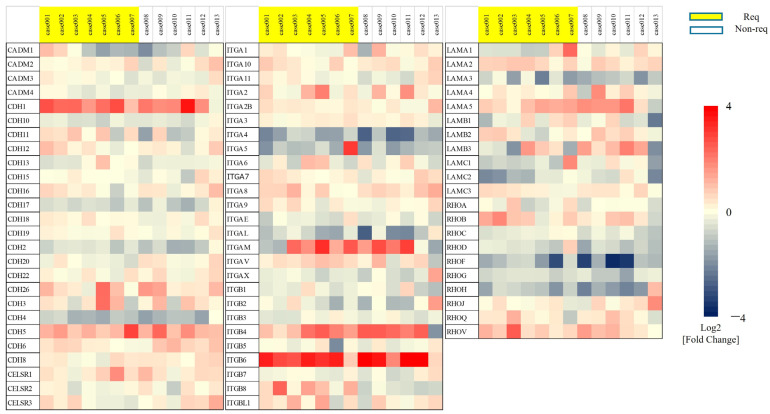
Microarray Analysis of Clinical Bladder Cancer Tissue—Extraction of Adhesion Factors. A total of 73 genes that encode proteins on the cell membrane surface and are directly involved in cell–cell adhesion were selected for analysis *Integrin β8* (*ITGB8*) was extracted as a gene significantly upregulated in the recurrence group compared with the non-recurrence group (0.95 ± 0.87 vs. −0.57 ± 0.27 log fold-change, *p* < 0.01).

Of 41 untreated patients with high-risk NMIBC, BCa tissue from 13 eligible patients obtained at the time of TURBT was used for the analysis. Background information pertaining to these 13 patients is shown in Table 1. There were 7 patients in the post-BCG recurrence group and 6 patients in the non-recurrence group. Of the 14,000 genes analyzed, 73 genes that encode cell membrane surface proteins directly involved in cell–cell adhesion were analyzed further. *Integrin β8* (*ITGB8*) was extracted as a gene significantly upregulated in the recurrence group compared with the non-recurrence group (0.95 ± 0.87 vs. −0.57 ± 0.27 log fold-change, *p* < 0.001, Figure 2). In this study, *ITGB8* was targeted for detailed analysis because of its increased expression in the recurrence group and decreased expression in the non-recurrence group compared with normal bladder tissue. Because the study cohort consisted of only 13 patients, multivariable analysis to assess whether ITGB8 expression had independent predictive value beyond standard clinical risk factors (stage, grade, CIS status, multiplicity, and tumor size) was not statistically feasible. Therefore, we performed descriptive comparisons of ITGB8 expression across these variables. For survival analysis, patients were stratified into high and low ITGB8 expression groups using a log2 fold-change cut-off of 0.5, which provided a biologically meaningful separation within the cohort (log-rank *p* < 0.01, Figure S1).

### 3.2. Suppression of ITGB8 Affects Cell Proliferation, Invasion, and Adhesion in Bladder Cancer Cells with High ITGB8 Expression

**Figure 3:**
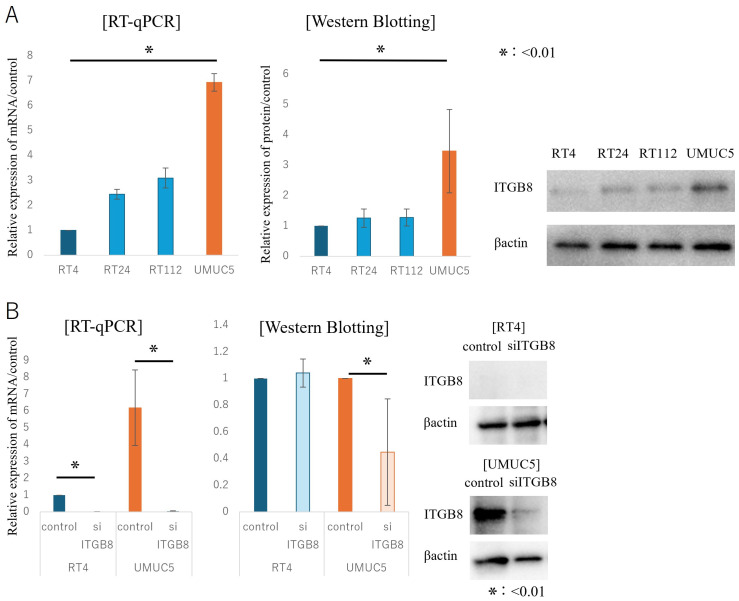
In Vitro Analysis of the Expression of ITGB8 in Human Bladder Cancer Cell Lines. (**A**) Analysis of the expression of ITGB8 in RT4, RT24, RT112, and UMUC5 human bladder cancer cells. (**B**) Expression of ITGB8 at the mRNA and protein levels was lowest in RT4 cells and highest in UMUC5 cells. Statistical significance was determined using an unpaired two-tailed Student’s *t*-test. The uncropped blots and molecular weight markers are shown in Supplementary Figure S2.

We evaluated RNA and protein expression of ITGB8 by RT-PCR and Western blotting (WB) in various human BCa cell lines, including RT4, RT24, RT112, and UMUC5. ITGB8 expression was lowest in RT4 cells and highest in UMUC5 cells (Figure 3).

**Figure 4:**
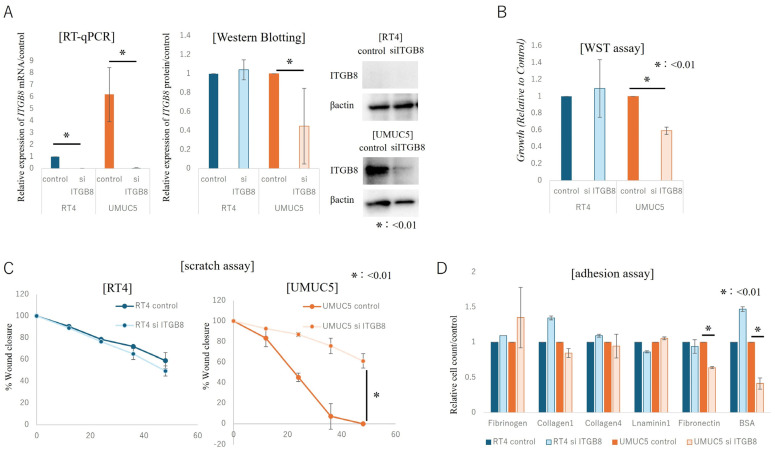
In Vitro Expression and Functional Analysis of ITGB8 With and Without Knockdown of *ITGB8*. Expression and functional analysis in RT4 human bladder cancer cells (low ITGB8 expression) and UMUC5 human bladder cancer cells (high ITGB8 expression) following knockdown of *ITGB8*. Analysis of mRNA and protein levels of ITGB8 in RT4 and UMUC5 cells before and after knockdown by quantitative reverse transcription–polymerase chain reaction (RT-qPCR) and Western blotting (**A**). Effects of ITGB8 knockdown on cell function (cell growth, invasion, adhesion) in both cell lines. Cell growth, invasion, and adhesion under small interfering RNA (siRNA)-induced *ITGB8* knockdown as determined by WST assay (**B**), scratch assay (**C**), and adhesion assay (**D**), respectively. Statistical significance was determined using an unpaired two-tailed Student’s *t*-test. The uncropped blots and molecular weight markers are shown in Supplementary Figure S3.

The effect of ITGB8 on cell function was examined by *ITGB8* knockdown using siRNA in two human BCa cell lines that differ in terms of ITGB8 expression: RT4 cells with low ITGB8 expression and UMUC5 cells with high ITGB8 expression. The effect of knockdown suppression on RNA and protein expression levels of ITGB8 was initially confirmed by RT-PCR and WB analyses in both cell lines. Following knockdown of *ITGB8*, expression of *ITGB8* RNA decreased in both cell lines; conversely, ITGB8 protein expression decreased only in UMUC5 cells, whereas no significant change was observed in RT4 cells (Figure 4A). The lack of response in ITGB8 protein expression in RT4 cells was attributed to the originally low expression of ITGB8 in these cells.

The WST assay was used to evaluate the effect of *ITGB8* knockdown on the proliferation of RT4 and UMUC5 cells. *ITGB8* knockdown significantly reduced the proliferation of UMUC5 cells (*p* < 0.001) but not that of RT4 cells (Figure 4B). Cell invasion potential was evaluated using a scratch assay. RT4 showed no significant change in invasiveness following *ITGB8* knockdown, whereas UMUC5 cells exhibited a significant decrease in invasiveness (*p* < 0.01, Figure 4C).

The effect of *ITGB8* knockdown on adhesion of UMUC5 and RT4 cells was also evaluated. The adhesion of RT4 cells to all extracellular matrices (fibrinogen, collagen 1 & 4, laminin 1, fibronectin, and control [BSA]) was not affected by *ITGB8* knockdown, but the adhesion of UMUC5 cells to fibronectin and BSA was significantly reduced (*p* < 0.01, Figure 4D).

In summary, *ITGB8* knockdown decreased cell proliferation, invasion potential, and adhesion (especially to fibronectin) only in UMUC5 cells, which typically express high levels of ITGB8. These results suggest that *ITGB8* regulates cell proliferation, invasion, and adhesion in human BCa cells that typically express high levels of ITGB8.

### 3.3. Suppression of TGF-β1 Secretion Following Knockdown of ITGB8 in Bladder Cancer Cells That Express High Levels of ITGB8

**Figure 5:**
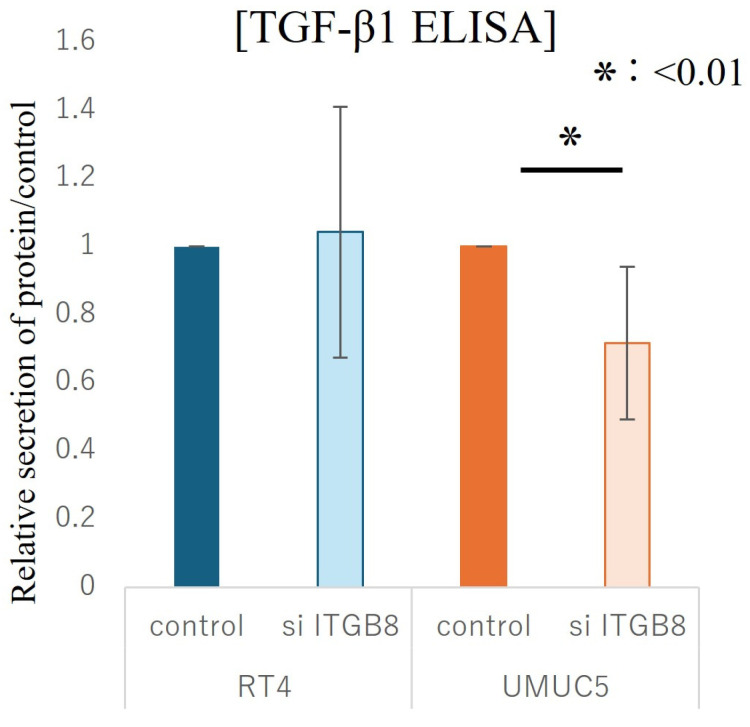
In Vitro Analysis of TGF-β1 Expression Following Knockdown of *ITGB8.* In RT4 cells and UMUC5 cells, secretion of TGF-β1 following siRNA-induced *ITGB8* knockdown was evaluated by ELISA. Statistical significance was determined using an unpaired two-tailed Student’s *t*-test.

The relationship between ITGB8 and the immunosuppressive factor TGF-β1 was evaluated in RT4 cells, which typically express low levels of ITGB8, and UMUC5 cells, which typically express high levels of ITGB8. After knockdown using either control siRNA or ITGB8 siRNA in both cell lines, the cells were cultured on plates supplemented with human fibronectin, and then TGF-β1 levels in the supernatant were measured using an ELISA. In low-ITGB8 RT4 cells, no significantly change in the concentration of TGF-β1 in the supernatant was observed between control siRNA and ITGB8 knockdown. By contrast, supernatant levels of TGF-β1 were significantly decreased in high-ITGB8 UMUC5 cells in which *ITGB8* was knocked down compared with control siRNA knockdown (*p* < 0.01, Figure 5). The results suggest that TGF-β1 secretion is regulated by ITGB8 in ITGB8-upregulated BCa cells.

### 3.4. ITGB8 Expression Is Correlated with Release of the Immunosuppressive Cytokine TGF-β1 in Bladder Cancer Tissue

**Figure 6:**
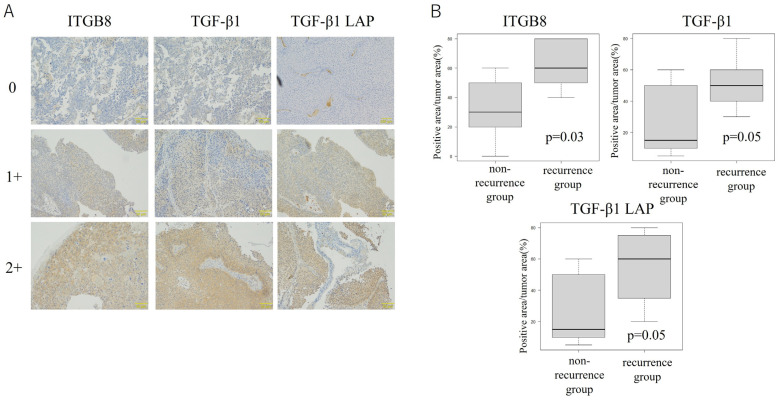
Analysis of ITGB8 and TGF-β1 Expression by Immunohistochemistry. The intensity of staining for TGF-β1, ITGB8, and TGF-β1 was classified into three levels: negative (0), weakly positive (1+), and positive (2+) (**A**). Immunohistochemistry of ITGB8, TGF-β1, and TGF-β1 LAP in tumor tissue at the time of transurethral resection of bladder tumor in the post-BCG recurrence group and the non-recurrence group (**B**). Inter-observer agreement of intensity scores was substantial (κ = 0.78), and discrepancies were resolved by consensus. Statistical significance was determined using an Mann–Whitney U test *t*-test.

To evaluate the relationship between ITGB8 and TGF-β1 and their distribution in the entirety of BCa specimens, immunohistochemistry using TGF-β1 LAP, which detects latent TGF-β1 as active, in addition to antibodies against ITGB8 and TGF-β1 was performed using specimens from the 7 patients in the recurrence group and 6 patients in the non-recurrence group. The intensity of TGF-β1, ITGB8, and latent TGF-β1 staining was classified as negative (0), weakly positive (1+), or positive (2+) (Figure 6A). In the recurrence group, a significantly greater area of the entire tumor was positive for ITGB8 (*p* = 0.03), TGF-β1 (*p* = 0.05), and TGF-β1 LAP (*p* = 0.05, Figure 6B) compared with the non-recurrence group. Thus, expression of both ITGB8 and TGF-β1 (which is potentially activated by ITGB8) was higher in the recurrence group than in the non-recurrence group.

## 4. Discussion

To our knowledge, this study provides the first evidence suggesting a potential role of ITGB8 in recurrence after BCG therapy in high-risk NMIBC. In addition, while TGF-β1 is well known to mediate immune suppression, our study offers novel insight by linking ITGB8 expression to TGF-β1 activation in the context of BCG-treated NMIBC.

BCG exerts its therapeutic effects: (1) direct anti-tumor effects of BCG against cancer cells [16,17], (2) induction of innate immune responses [10,18,19], and (3) adaptive immune responses [10]. Thus, BCG must induce an effective immune response; however, the immune induction by BCG varies from case to case [7,8,16,19]. This difference is thought to be influenced by factors on the host (patient) and tumor sides. Host-side factors that modulate immune response include differences in individual immune responses to BCG infection [7,8,16,20]. As shown by the tuberculin response to BCG, even among healthy individuals, the response to BCG can vary depending on several factors, and similar individual differences are expected in patients with BCa. Furthermore, even if immunity is successfully induced, the immune response can be suppressed through a mechanism known as “immune escape” or “immunosuppression”, leading to recurrence [13]. Immunosuppressive factors such as TGF-β1 [21] and PD-L1 [22] reportedly promote recurrence in NMIBC post-BCG. In particular, the immunosuppressive cytokine TGF-β1 has the potential to “trigger” escape from the anti-tumor immunity induced in the bladder by BCG treatment.

In this study, ITGB8 was highly expressed in patients who experienced recurrence compared with patients who did not experience recurrence. This suggests that ITGB8 expression is highly upregulated in patients in whom BCG treatment induces appropriate immunity, but the immune response is suppressed due to immune escape. ITGB8 first binds to the RGD region of the ECM when cells adhere to the bladder [23,24]. When ITGB8 binds to the RGD region, proteases are secreted that cleave the TGF-β1 and LAP region boundary in the latent TGF-β1 in the stroma and cells, resulting in secretion of active TGF-β1 [24]. In this study, knockdown of *ITGB8* in a human BCa cell line with high ITGB8 expression suppressed not only cell proliferation, invasiveness, and adhesion to fibronectin but also secretion of the immunosuppressive cytokine TGF-β1. In addition, immunohistochemistry of tumor tissue showed higher expression of ITGB8, TGF-β1, and TGF-β1 LAP, which indicates latent TGF-β1 activation in recurrent cancer tissues compared with non-recurrent cancer tissues after BCG treatment. These results thus suggest that ITGB8 regulates immunosuppression in BCa cells by promoting the release of TGF-β1 via adhesion to fibronectin. Although these findings support the biological plausibility that ITGB8 activates latent TGF-β1 and promotes an immunosuppressive microenvironment, our study does not fully delineate the downstream immune modulatory mechanisms. Previous reports have demonstrated that integrin αvβ8 directly activates latent TGF-β1 through cleavage of the LAP domain and plays a central role in immunosuppression and tumor immune escape in several cancer types [9,24]. Furthermore, αvβ8-mediated TGF-β1 activation has been shown to contribute to regulatory T-cell induction and suppression of antitumor immunity [25]. In this study, the increase in ITGB8, TGF-β1, and TGF-β1 LAP expression in recurrent tissues, together with the reduction in TGF-β1 secretion after ITGB8 knockdown in vitro, is consistent with these established mechanisms. However, direct functional evidence that ITGB8-driven TGF-β1 activation leads to immune escape in the context of BCG therapy remains to be established. Specifically, immune cell-based assays or BCG-stimulated functional studies would be required to confirm the immunological consequences of ITGB8 activity. These mechanistic experiments represent an important direction for future research.

In this study, all patients had received BCG treatment, and the disappearance of tumors at least once was confirmed by cystoscopy and cytology. Therefore, initial intravesical BCG treatment was considered to induce adequate anti-tumor immune responses. In cases of recurrence, ITGB8-positive BCa cells that survive due to immune escape secrete immunosuppressive cytokines, suggesting that they could have escaped the anti-tumor immunity induced by BCG treatment and recur intravesically. Thus, in ITGB8-positive BCa, it is thought that suppression of *ITGB8* could inhibit recurrence.

The present study has some limitations. First, it is possible that tumors could have been completely resected through transurethral resection in non-recurrence patients. Second, the relatively low number of cases included in the study presents some limitations. Although ITGB8 expression was associated with recurrence in our study, the small sample size precluded reliable multivariable modeling to establish whether ITGB8 has independent predictive value beyond conventional clinical factors such as stage, grade, CIS, multiplicity, and tumor size. Therefore, our findings should be interpreted as preliminary and hypothesis-generating. Nevertheless, the clear separation in recurrence-free survival between high and low ITGB8 groups, defined using a log2 fold-change cut-off of 0.5, supports the potential prognostic value of ITGB8 expression. Validation in larger, independent cohorts will be essential to confirm its clinical utility. Furthermore, because early BCG failures were excluded, the findings may apply primarily to mid- to long-term recurrence, limiting broader clinical applicability. Third, to control for factors that contribute to discontinuation due to surgical manipulation or side effects, other than the therapeutic effect of BCG, patients who recurred immediately after initiation of BCG treatment or who discontinued BCG were excluded from this study. This potentially excluded cases with strong resistance to BCG. Due to these limitations, we believe that further verification involving studies with more cases is needed. Finally, this study is limited by the small and highly selected cohort. To focus on patients with high-risk NMIBC who completed induction BCG therapy and underwent long-term follow-up, 28 of the 41 initially screened patients were excluded. This selection process may have introduced selection bias, potentially enriching the cohort for a specific biological phenotype. Therefore, the findings should be interpreted with caution and should be validated in larger and less selected patient populations.

Our study suggests that high ITGB8 expression is associated with mid- to long-term intravesical recurrence in high-risk NMIBC. Our results also suggest that ITGB8 is associated with the release of TGF-β, in turn suggesting that adhesion molecule-mediated immunosuppression may contribute to recurrence. Evaluations of ITGB8 may thus be useful for predicting mid- to long-term recurrence in NMIBC. In the future, we will evaluate the usefulness of immunohistochemistry of ITGB8 tumor tissues in predicting BCG treatment efficacy.

## 5. Conclusions

In this study, we demonstrated that high ITGB8 expression is associated with increased TGF-β1 activation and with recurrence in patients with high-risk NMIBC following BCG therapy. Our in vitro findings further support the role of ITGB8 in promoting an immunosuppressive tumor environment through the release of active TGF-β1. Although the cohort size was limited and early BCG failures were not included, these results suggest that ITGB8 may contribute to immune escape and could serve as a potential biomarker for identifying patients at elevated risk of recurrence. Further studies in larger and more diverse patient populations, including functional assays of BCG-induced immunity, will be necessary to validate the clinical utility of ITGB8.

## Figures and Tables

**Table 1:** Patient Characteristics.

	Total (n = 13)	Recurrence Group (*n* = 7)	Non-Recurrence Group (*n* = 6)	
age(average)	73.5 ± 8.5	72.1 ± 9.1	73.5 ± 9.7	n.s
sex (male:female)	11:2	7:0	4:2	n.s
cytology (positive:negative)	9:4	5:2	3:3	n.s
tumor diameter(average)	20.0 ± 7.3	22.5 ± 7.4	22.7 ± 6.4	n.s
pathological grade (high:low)	4:9	3:4	1:5	n.s
pT stage (pTa:pT1)	11:2	6:1	5:1	n.s
Cis (with or without)	1:12	1:6	0:6	n.s

## Data Availability

The original contributions presented in this study are included in the article/supplementary material. Further inquiries can be directed to the corresponding author.

## Supplementary Materials

The following supporting information can be downloaded at: https://www.mdpi.com/article/10.3390/cancers17243964/s1, Figure S1: Kaplan–Meier curves stratified by ITGB8 expression (high vs. low); Figure S2: The uncropped blots of Figure3; Figure S3: The uncropped blots of Figure 4; Table S1: Characteristics of Excluded Patients.

## Abbreviations

BCG	Bacillus Calmette–Guérin
cDNA	complementary DNA
ELISA	enzyme-linked immunosorbent assay
mRNA	messenger RNA
PD-L1	programmed cell death ligand 1
RT-PCR	reverse transcription–polymerase chain reaction
siRNA	small interfering RNA or short interfering RNA
TGF-β1	transforming growth factor–β
