# Supplementary material for: Immunosuppressive Role of Integrin β8 in Recurrence After Bacillus Calmette–Guérin (BCG) Therapy for Non-Muscle Invasive Bladder Cancer
Source: Cancers (Basel). 2025 Dec 12;17(24):3964. doi: 10.3390/cancers17243964 (PMC12730356; doi:10.3390/cancers17243964)
Supplement: Supplementary file 1 [file cancers-17-03964-s001.zip › TableS1.pdf]

TableS1: Characteristics of Excluded Patients

	Total (n=28)	relapsed within 6 times BCG patients (n=8)	Dropped out of BCG patients (n=15)	lost to follow-up (n=5)
age(average)	72.4±8.3	69.0±3.7	73.5±9.7	74.0±9.5
sex (male : female)	21:07	6:2	11:4	4:1
cytology (positive : negative)	9:19	6:1	2:14	1:4
tumor diameter(average)	17.3±9.5	17.5±8.5	22.7±6.4	19.1±11.1
tumor number(average, range)	2 (1-11)	5 (2-11)	2(1-4)	2(1-2)
pathological grade (high : low)	12:16	8:0	3:12	1:4
pT stage (pTa : pT1)	20:08	4:4	13:2	3:2
Cis (with or without)	2:26	1:7	1:14	0:5
duration of follow up (day, average)	2733±1672.5	3902.2±1205.5	2164.1±373.3	864±164.3
number of recurrent patients	10	8	2	0
time to recurrence	N/A	66.8±8.7	779±175.3	N/A
