# Supplementary figures and images for: Immunosuppressive Role of Integrin β8 in Recurrence After Bacillus Calmette–Guérin (BCG) Therapy for Non-Muscle Invasive Bladder Cancer
Source: Cancers (Basel). 2025 Dec 12;17(24):3964. doi: 10.3390/cancers17243964 (PMC12730356; doi:10.3390/cancers17243964)

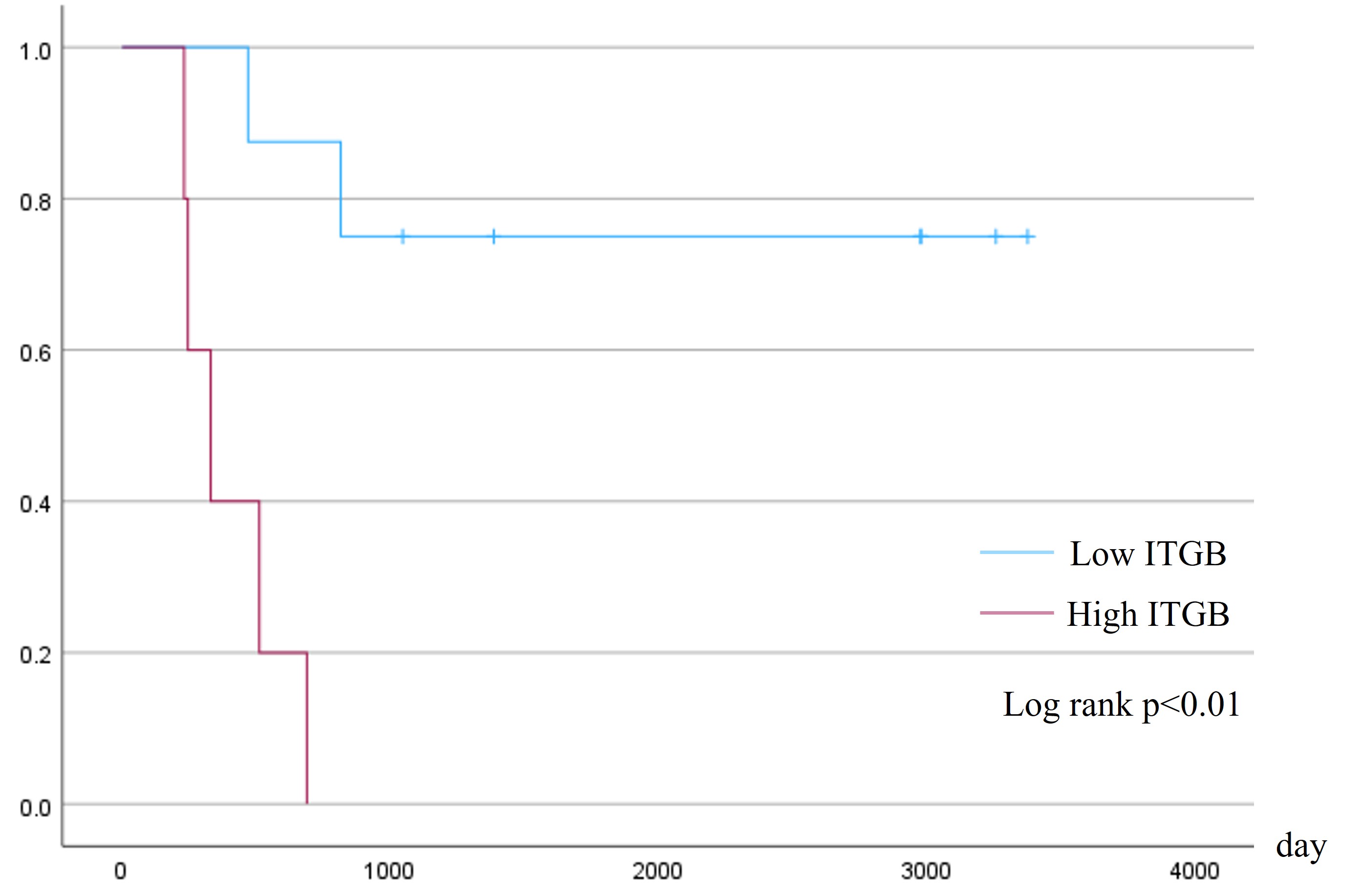

Supplement: Supplementary file 1 [file cancers-17-03964-s001.zip › FigureS1.jpg]

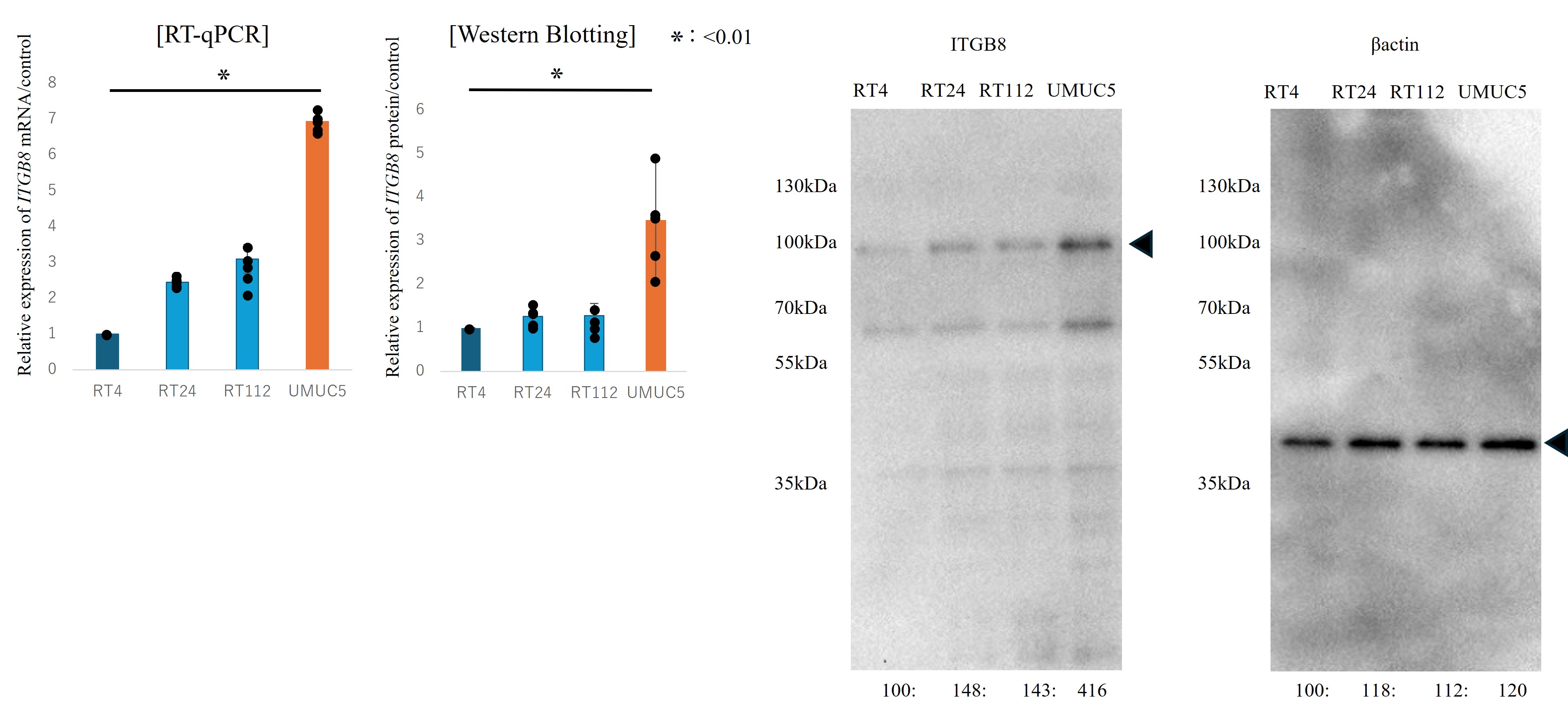

Supplement: Supplementary file 1 [file cancers-17-03964-s001.zip › FigureS2.jpg]

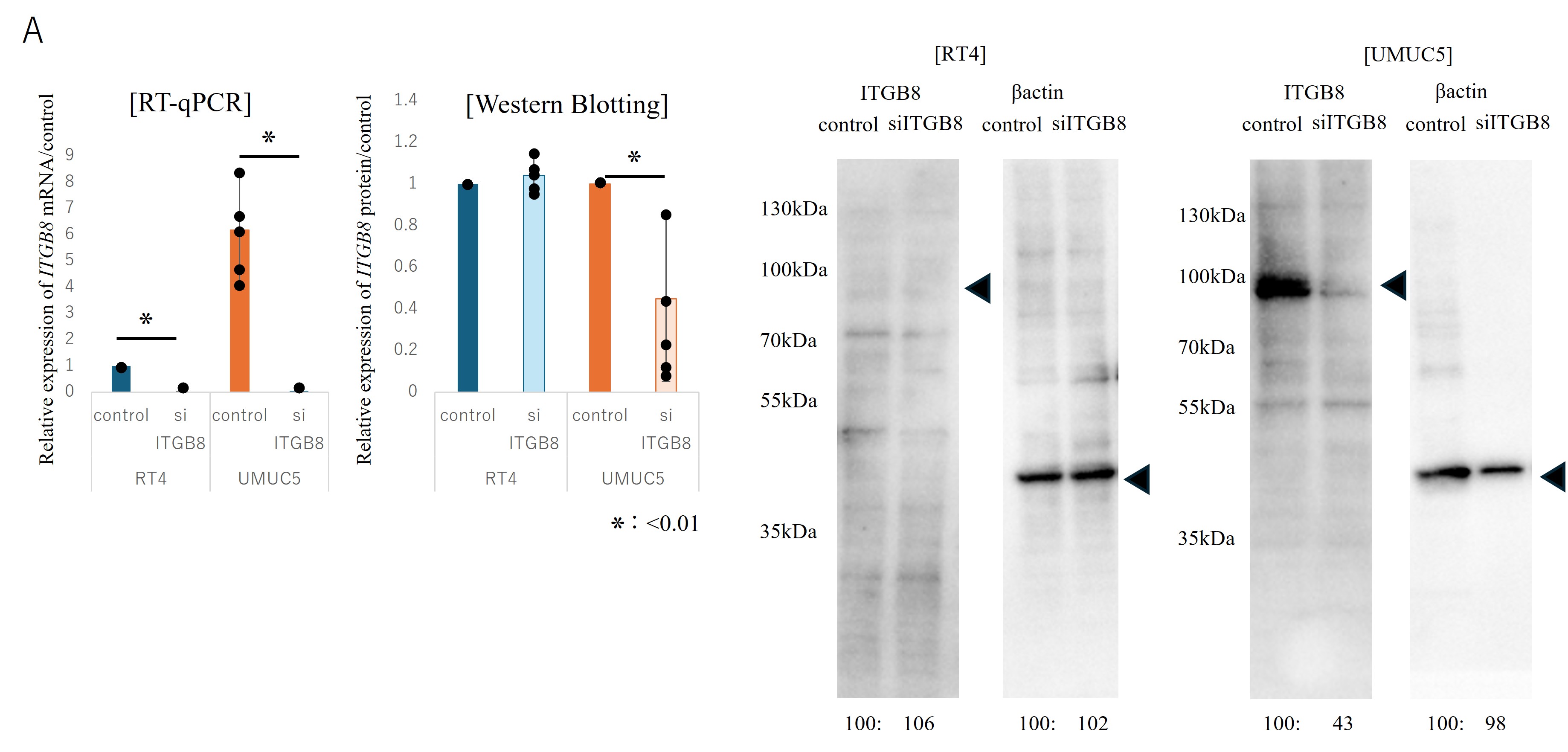

Supplement: Supplementary file 1 [file cancers-17-03964-s001.zip › FigureS3.jpg]
